# Segmentation of Young Polish Consumers in Relation to Product Attributes and Willingness to Consume Food Containing Edible Insects

**DOI:** 10.3390/insects16090980

**Published:** 2025-09-19

**Authors:** Anna Platta, Anna Mikulec, Monika Radzymińska, Karolina Mikulec, Stanisław Kowalski

**Affiliations:** 1 Faculty of Management and Quality Science, Gdynia Maritime University, 81-87 Morska Street, 81-225 Gdynia, Poland; a.platta@wznj.umg.edu.pl; 2 Faculty of Engineering Sciences, University of Applied Science in Nowy Sacz, 1A Zamenhofa Street, 33-300 Nowy Sacz, Poland; amikulec@ans-ns.edu.pl; 3 Faculty of Economic Sciences, Institute of Management Science and Quality, University of Warmia and Mazury in Olsztyn, 4 Oczapowskiego Street, 10-719 Olsztyn, Poland; mradz@uwm.edu.pl; 4 Graduate of the Warsaw School of Economics, 162 Niepodległości Avenue, 02-554 Warsaw, Poland; karolinaa.mikulec@gmail.com; 5 Department of Carbohydrate Technology and Cereal Processing, Faculty of Food Technology, University of Agriculture in Krakow, 122 Balicka Street, 30-149 Krakow, Poland

**Keywords:** novel food, factors of choice, sustainable consumption, environmental sustainability, food neophobia, Generation Z

## Abstract

This study investigated consumer segmentation regarding insect-based foods among Polish Generation Z students. Data from 947 respondents were analyzed using exploratory factor analysis (EFA) followed by k-means clustering. Two segmentation solutions were identified: a four-cluster model based on product categories and a three-cluster model based on product attributes. In the four-cluster solution, the segments were labeled insect product enthusiasts, pragmatic consumers of processed products, cautious explorers, and skeptical and disengaged. In the three-cluster solution, the clusters were identified as conscious enthusiasts, quality-oriented pragmatists, and skeptical and disengaged. Across both approaches, the clusters differed significantly in terms of food neophobia, perceptions of sustainability, and product-related attributes such as safety, nutritional value, and sensory appeal. Notably, socio-demographic factors such as gender, place of residence, economic status, and field of study did not show statistically significant differences across clusters. Food neophobia emerged as a strong and highly significant differentiator (*p* < 0.001), reinforcing its role as a major psychological barrier, while positive perceptions of safety, taste, and nutritional value enhanced acceptance. These findings extend current knowledge on consumer heterogeneity in sustainable food markets and provide practical guidance for developing tailored communication strategies to promote the acceptance of edible insects among young consumers.

## Simple Summary

Eating insects is often presented as a sustainable way to produce protein, but in many European countries, people are still hesitant to include them in their diets. In this study, we looked at how young Polish consumers, especially university students, view insect-based foods. We found that young people are not all the same: some are enthusiastic about trying insect products, others are curious but cautious, while many remain skeptical or uninterested. What mattered most about these differences were personal attitudes and perceptions. Students who were less afraid of trying new foods and who cared about the environment were more open to eating insects. In contrast, fear of unfamiliar foods, doubts about safety, and feelings of disgust created strong barriers. Interestingly, factors such as gender, place of residence, or field of study did not make a clear difference. These results show that communication should focus on building trust, highlighting taste and nutritional value, and providing clear information about safety in order to make insect-based foods more acceptable.

## 1. Introduction

The contemporary world faces numerous challenges related to food security, environmental degradation, and climate change. The projected global population increase to 9.7 billion by 2050 [1], combined with the depletion of natural resources and greenhouse gas emissions from intensive livestock farming, necessitates the search for alternative, more sustainable sources of protein [2,3]. In this context, entomophagy, the practice of consuming insects, is gaining attention for its potential to support more sustainable food systems, owing to the high nutritional value of edible insects, their low greenhouse gas emissions, and minimal resource requirements [4,5,6].

While entomophagy is common in some parts Asia, Africa, and Latin America, it continues to face significant psychological and cultural barriers in Europe [7,8]. In Western societies, feelings of disgust and food neophobia present major obstacles to the integration of insects into the human diet [9,10,11,12].

Despite the growing body of international research, in-depth analyses focused on Poland remain scarce, both in cultural and psychographic terms. Little is known about the relationships between personality traits, pro-environmental values, and attitudes toward insects as a food source. The present study seeks to address this gap by investigating the determinants of willingness to consume insect-based foods among young Polish adults. The study integrates psychological, perceptual, and sociodemographic predictors and applies consumer segmentation to identify distinct profiles of respondents.

Previous studies highlight the importance of psychological, perceptual, and sociodemographic factors in shaping consumer acceptance of insect-based foods [13].

Gender consistently emerges as a determinant, with men more willing than women to accept insects as food [14,15]. Age-related differences are also reported in Europe, where younger consumers show higher openness compared with older generations [16,17,18]. However, research in Poland suggests that consumer knowledge of insect-based foods remains limited and no significant age effects were observed [19]. Among Generation Z students, acceptance was linked with pleasure orientation in men, low attachment to culinary traditions in both genders, and health-related motives in women [20].

Education and place of residence further contribute to acceptance. Higher educational attainment is associated with openness to novel dietary concepts and stronger appreciation of sustainability and nutritional value [10,21,22,23]. Urban residents tend to be more receptive, although acceptance remains limited where insect consumption is not rooted in culinary traditions [24,25]. In Poland, reluctance and concerns about insect-based foods were influenced by residence, income, and education, which also shaped perceptions of health and environmental benefits [19].

Beyond sociodemographic influences, psychological factors such as food neophobia act as barriers, while environmental concern supports openness to alternative proteins [26,27,28,29,30,31,32]. Product-specific attributes, including sensory appeal, nutritional value, safety, and origin, have also been shown to influence consumer intentions [33,34].

Based on these insights, six hypotheses were proposed.

**H1:**  *Gender differences were expected to significantly influence individuals’ willingness to include edible insects in their diet.*

**H2:**  *Food neophobia was assumed to act as a psychological barrier, reducing acceptance of insect-based foods.*

**H3:**  *Concern for environmental sustainability was regarded as a motivating factor likely to increase openness to consuming insects.*

**H4:**  *Socio-demographic characteristics such as place of residence, self-assessed economic status, and study profile were expected to differentiate consumer groups regarding insect consumption.*

**H5:**  *Perceptions of product-specific attributes such as nutritional value, sensory appeal, safety, and origin were predicted to play an important role in shaping intentions toward insect-based products.*

**H6:**  *Distinct consumer segments would emerge among young Polish adults, each characterized by varying levels of acceptance of edible insects in modern diets.*

## 2. Materials and Methods

### 2.1. Data Collection and Sample Characteristics

The segmentation of young consumers was based on data collected through a study conducted among students enrolled in higher education institutions in Poland. Only students who reported consuming all types of food, without restricting their intake of meat or animal products, were eligible to participate. The study protocol was approved by the University Ethics Committee for Scientific Research at the Krakow University of Economics (Approval No. KEBN/71/0044/D24/2023). The list of survey questions used in this study is available in the Supplementary Material (S1_Survey_Questions). A non-probability purposive sampling method was used, and participation was voluntary. All respondents provided informed consent after being briefed on the purpose of the research and the potential risks associated with the Computer-Assisted Web Interviewing (CAWI) procedure. The questionnaire was distributed through official university channels, including the e-learning platform, Microsoft Teams, and direct communication from instructors during classes. No incentives were offered for participation. Because the survey required respondents to complete all items before moving to the next section, the dataset contained no missing values, and neither imputation nor case exclusion was necessary. The research instrument was a structured questionnaire administered online using the CAWI method. It was divided into two main sections. The first section captured quantitative variables, including the following:

Concern for food safety (CFS), measured using the scale developed by Mikulec et al. [13];
Concern for environmental sustainability (CES), also following Mikulec et al. [13];
Food neophobia (FN), assessed with the scale by Pliner and Hobden [26].

Willingness to consume novel food products containing edible insects in various forms (fresh, frozen, dried, powdered, e.g., insect flour), across 11 product categories: burgers and processed meat products; ready-to-eat meals (e.g., soups, pasta dishes, pancakes); bakery products (e.g., bread, rolls, pizza); confectionery (e.g., cakes, cookies, chocolate-covered insects); snacks (e.g., bars, crisps); post-workout beverages and high-protein supplements; dairy-based products (e.g., cottage cheese, yogurt, milk drinks); sauces and mayonnaise; cricket (frozen, dried, or powdered); mealworm larvae (frozen, dried, or powdered); migratory locust (frozen, dried, or powdered) [35]. Additionally, the survey assessed the importance of 13 product attributes in influencing purchase decisions for insect-based novel foods: appealing taste; pleasant aroma; attractive appearance; high nutritional value; product variety and market availability; popularity (trendiness); affordable price; package size and attractiveness; ease of use; health claims; nutritional claims; reduction in CO_2_ emissions; availability of recipes on blogs and food-related websites [12].

During the survey, the respondent expressed his or her level of approval or disapproval of all the posted items using a 5-point Likert scale, where response options were: 1—“definitely no”, 2—“no”, 3—“I do not know, I have no opinion”, 4—“yes”, 5—“definitely yes” [36].

The assessment of willingness to consume novel food products containing edible insects, as well as the perceived importance of specific product attributes, was structured around latent dimensions identified through exploratory factor analysis (EFA), which were subsequently used as the basis for consumer segmentation.

The second part of the questionnaire included a demographic section, which captured qualitative variables related to the respondents, such as gender, place of residence, and self-reported assessment of their financial situation.

The collected empirical data were subjected to statistical analysis, including the assessment of scale reliability using Cronbach’s alpha coefficients, as recommended by Chan and Idris [37]. A minimum acceptable alpha value of 0.70 was applied as the criterion for internal consistency. All scales used in the study demonstrated high internal consistency, exceeding the generally accepted threshold for satisfactory reliability (α > 0.70) [38]. Specifically, Cronbach’s alpha was 0.92 for the Concern for Food Safety scale (CFS), 0.88 for the Concern for Environmental Sustainability scale (CES), and 0.80 for the Food Neophobia scale (FN), indicating robust psychometric properties across all constructs measured.

The primary aim of this study was twofold: (a) to examine the influence of demographic, psychological, and perceptual predictors on young consumers’ willingness to consume foods containing edible insects (H1–H5), and (b) to identify distinct consumer segments within this population (H6).

### 2.2. Statistical Analyses

#### 2.2.1. Exploratory Factor Analysis

To identify the latent dimensional structure of the 11 evaluated product categories, an exploratory factor analysis (EFA) with Oblimin rotation was applied. Sampling adequacy was confirmed by a high Kaiser–Meyer–Olkin (KMO) value of 0.935, with all Measures of Sampling Adequacy (MSA) for individual variables ranging from 0.878 to 0.960, indicating very good fit to the factor model. Bartlett’s test of sphericity was significant (χ^2^(55) = 12,328; *p* < 0.001), supporting the appropriateness of dimension reduction. The analysis revealed three moderately correlated factors (r = 0.479–0.502), suggesting three interrelated dimensions underlying consumer perceptions of the selected product categories.

A second EFA was then conducted for the 13 product attributes related to edible insects, again using Oblimin rotation. Sampling adequacy was excellent, as indicated by a very high KMO value of 0.951, with all MSA values exceeding 0.90. Bartlett’s test of sphericity was significant (χ^2^(78) = 15,278; *p* < 0.001), confirming the validity of factor extraction. This analysis also identified three moderately correlated factors (r = 0.453–0.489), which describe distinct but interrelated dimensions of consumer perceptions of product attributes. In both analyses, only factor loadings ≥ 0.40 were retained, in line with methodological recommendations that this threshold ensures interpretability of the extracted factors [39,40].

#### 2.2.2. Cluster Analysis

Factor scores were computed as weighted means of *z*-scored items, using the factor loading matrix as weights so that items with higher loadings contribute more to each score. The three factor scores provide a low-dimensional, noise-reduced representation of consumer perceptions. Before clustering, the factor scores were standardized (*z*-scores) to place them on a common scale, preventing any factor from dominating due to variance differences.

Cluster robustness was assessed with a resampling procedure. In 100 iterations, 80% of respondents were randomly selected without replacement, k-means (k = 4) was refitted, and the resulting labels were compared with the reference solution using the Adjusted Rand Index (ARI). In addition, for each candidate solution (k = 2–10), we computed the proportion of variance explained (PVE = 1 − WSS/TSS, where WSS is the within-cluster sum of squares and TSS the total scatter around the global mean), along with standard validity indices (Silhouette coefficient, Calinski–Harabasz index, Davies–Bouldin index). These complementary measures supported the use of the elbow method to determine the optimal number of clusters, selecting the smallest k beyond which additional clusters yielded only marginal reductions in within-cluster variance.

Using factor scores instead of raw mean ratings improved both stability and interpretability, enabling clusters to be profiled in terms of high, medium, or low scores along latent dimensions rather than relying on multiple individual items. In the description of clusters, qualitative terms such as “moderate” or “average” were used to denote mid-range values, i.e., factor scores close to zero after z-standardization or mean values located around the midpoint of the 5-point Likert scales.

Psychological and sociodemographic variables were applied solely for post hoc characterization of the clusters, rather than being incorporated into the clustering procedure itself.

#### 2.2.3. Testing Differences Between Consumer Clusters

Nevertheless, Kruskal–Wallis tests were also conducted as a nonparametric robustness check, and the results were consistent with the ANOVA outcomes, supporting the validity of the reported findings. For categorical variables (gender, field of study, place of residence, and self-declared economic status), chi-square tests of independence were performed. To complement significance testing, Cramér’s V was reported as an effect size measure, indicating the strength of associations between cluster membership and each categorical variable.

Because multiple comparisons were performed, *p*-values from both chi-square tests and post hoc analyses were adjusted using the Benjamini–Hochberg (BH) correction to control the false discovery rate. This approach balances error control with sufficient power to detect meaningful differences in exploratory consumer segmentation studies.

All analyses were performed using Statistica v. 13.0 (StatSoft, Tulsa, OK, USA), and Python (version 3.11) with standard scientific libraries, including pandas, numpy, scikit-learn, scipy, and statsmodels.

## 3. Results

The study sample consisted of 947 young adults enrolled in higher education institutions in Poland. Table 1 presents the sociodemographic profile of the respondents. Overall, the sample was balanced in terms of gender, with women slightly outnumbering men. The largest groups of students represented engineering/technical sciences and social sciences. Most respondents lived in rural areas or medium-sized cities, and the majority assessed their economic status as good or sufficient.

To enhance the clarity of statistical analyses and ensure sufficient sample sizes within comparative subgroups, selected categorical variables were consolidated. Regarding the field of study, disciplines with relatively small representation were grouped into two broader categories: “natural sciences,” encompassing medical and health sciences, agricultural sciences, as well as natural and physical sciences, fields unified by their empirical orientation and practical applications; and “social and humanities sciences,” a combined category including social and humanistic disciplines, characterized by their shared focus on societal, cultural, and psychological phenomena. For the variable related to place of residence, respondents living in cities with populations between 150,000 and 500,000 and those from cities with more than 500,000 inhabitants were combined into a single category, urban areas above 150,000 residents, due to their comparable urban character and to promote more balanced group sizes. Similarly, economic status categories were aggregated to improve analytical clarity. Participants who self-reported their financial situation as “insufficient” or “sufficient” were classified as belonging to the “lower economic status” group, while those who rated their situation as “very good” or described themselves as having an exceptionally high economic status were grouped into the “higher economic status” category. This methodological approach to variable aggregation was intended to facilitate more consistent interpretation of the results and to reduce statistical noise associated with the presence of small, heterogeneous subgroups.

Following these adjustments, 43.50% of the respondents were enrolled in programs categorized as social and humanities sciences, 41.08% in engineering and technical sciences, while 15.42% studied within the natural sciences domain. With respect to residence, the largest share of respondents lived in rural areas (36.85%). A further 17.63% resided in small towns with up to 50,000 inhabitants, while 16.58% were from medium-sized towns (50,000–150,000 inhabitants). With respect to residence, 28.94% of participants resided in larger urban centers (i.e., cities with populations exceeding 150,000). In terms of subjective economic status, 25.55% of respondents were classified as having a lower economic status, 53.01% as having a moderate (good) status, and 21.44% as belonging to the higher economic level.

### Exploratory Factor Analysis

To further explore consumer perceptions, two separate exploratory factor analyses (EFAs) were conducted. The first focused on product categories containing edible insects, and the second examined food attributes associated with these products.

The analysis of product categories revealed a three-factor solution explaining 83.1% of the variance (Table 2).

The first factor grouped together conventional and easily recognizable products, such as burgers and processed meat products, ready-to-eat meals, bakery items, confectionery, and snacks. With the exception of post-workout beverages and high-protein supplements, the loadings in this dimension were high (0.809–0.931), supporting its interpretation as “conventional food products” (Table 3). The second factor encompassed insects presented in frozen, dried, or powdered form (cricket, mealworm larvae, and migratory locust). Loadings in this factor were all above 0.890, highlighting its unambiguous character, and it can therefore be described as “insect-based products”. The third factor related to dairy-based and complementary culinary products, with a very high loading for dairy (0.982). This dimension is best labeled as “dairy and culinary add-ons” (Table 3).

The second EFA, conducted on food attributes, also revealed a three-factor solution, jointly explaining 79.6% of the variance (Table 4).

The first factor, interpreted as a “sensory–economic” dimension, included attributes related to taste, aroma, appearance, and price, with factor loadings ranging from 0.478 to 0.933 (Table 5). The second factor reflected a “practical-market” dimension, capturing aspects of availability, ease of use, popularity, packaging, environmental considerations, and the availability of recipes online (factor loadings between 0.523 and 0.754). The third factor was associated with “informational-health” attributes, specifically health and nutrition claims, which showed the highest loadings (above 0.930) (Table 5). Such high loadings indicate a particularly consistent consumer perception in this area, strongly oriented toward producers’ declarative information regarding health and nutritional value.

## 4. Consumer Segmentation

Consumer segmentation was conducted on the basis of latent dimensions (factors) derived from exploratory factor analysis (EFA). Two separate clustering procedures were performed. In the first analysis, three factors: conventional products, insect-based products, and dairy and culinary add-ons were used as input for a *k*-means cluster analysis, which identified a four-cluster solution as the most appropriate. In a parallel analysis focusing on product attributes, three latent dimensions: sensory–economic, practical–market, and informational–health were extracted, and a *k*-means procedure revealed a three-cluster solution. Psychological and sociodemographic variables were used solely for post hoc profiling of the identified clusters, rather than as inputs to the clustering procedure.

### 4.1. Consumer Segmentation Based on EFA Factors Related to Willingness to Consume Insect-Based Foods

Cluster analysis using the k-means method indicated that a four-cluster solution was the most appropriate (Table 6). Cluster sizes were well balanced overall, with Cluster 1 comprising 295 respondents (31.15%), Cluster 2 including 252 (26.61%), Cluster 3 including 282 (29.78%), and Cluster 4 including 118 (12.46%). Cluster reproducibility was evaluated through repeated subsampling: across 100 runs with 80% of the data, the Adjusted Rand Index (ARI) averaged at 0.92, with the 2.5–97.5% quantiles ranging from 0.56 to 1.00. These values indicate good but somewhat variable stability, suggesting that while the overall cluster structure is robust, the allocation of a minority of individuals may fluctuate across resamples. For k = 4, the within-cluster sum of squares (WSS = 628.1) relative to the total scatter (TSS = 2841.0) yielded a proportion of variance explained of PVE = 0.78, showing that the model accounted for a substantial share of variability in the data. Complementary validity indices were consistent with an adequate solution (Silhouette coefficient = 0.48, Calinski–Harabasz index = 1107.5, Davies–Bouldin index = 0.99). Overall, the clustering revealed four coherent and interpretable groups, with statistical indicators supporting both the internal consistency and external validity of the segmentation.

#### 4.1.1. Cluster 1—Pragmatic Consumers of Processed Products

Respondents in this cluster reported high acceptance of conventional food products (F1 = 0.86) and moderate acceptance of dairy and culinary add-ons (F3 = 1.02), while their acceptance of insect-based products remained low (F2 = −0.36) (Table 6). Psychologically, they were characterized by low food neophobia (NF = 1.89) and a high level of concern for food safety (CFS = 3.73) (Table 7). Women accounted for 66.14% of this cluster (Table 8). Respondents lived both in rural areas (33.86%) and in large cities with more than 150,000 inhabitants (32.28%). A majority reported a moderate economic status (59.84%) (Table 8). In terms of academic profile, 45.67% were students of social and humanities sciences (Table 8).

#### 4.1.2. Cluster 2—Skeptical and Disengaged

This group consistently scored low across all factors (Table 6), indicating clear reluctance toward insect-based products. Psychologically, they exhibited the highest level of food neophobia (NF = 2.56) and the lowest concern for both food safety (CFS = 2.42) and environmental sustainability (CES = 2.31) across the clusters (Table 7). Women made up 62.95% of this cluster (Table 8). The largest share of respondents resided in rural areas (38.86%). Economically, 25.60% reported lower and 24.10% higher economic status. Regarding the field of study, 40.96% were students of engineering and technical sciences, while 43.98% studied social and humanities sciences (Table 8).

#### 4.1.3. Cluster 3—Insect Product Enthusiasts

This was the most open segment toward insect-based foods, recording the highest scores across all three factors (Table 6). They displayed low food neophobia (NF = 1.99), strong concern for food safety (CFS = 4.09), and high pro-environmental attitudes (CES = 4.03) (Table 7). Women represented 56.13% of this cluster (Table 8). The majority of respondents resided in rural areas (33.55%) and large cities (30.97%). A moderate economic status was most frequently declared (50.32%). In terms of field of study, 41.94% were enrolled in engineering and technical sciences (Table 8).

#### 4.1.4. Cluster 4—Cautious Explorers

Respondents in this cluster showed moderate factor scores (Table 6), suggesting a cautious but not entirely negative attitude toward insect-based products. They exhibited medium levels of food neophobia (NF = 2.19), and moderate concern for both food safety (CFS = 3.33) and environmental sustainability (CES = 3.15) (Table 7). Women accounted for 56.46% of the group (Table 8). The largest proportions of respondents came from rural areas (37.54%) and large cities (30.03%). A majority reported a moderate economic status (54.35%). With regard to the field of study, 45.35% were students of social and humanities sciences (Table 8).

Table 8 presents the distribution of sociodemographic characteristics across clusters.

#### 4.1.5. Factors Differentiating Product-Based Clusters

Chi-square tests and ANOVA confirmed that the most pronounced differences between clusters were observed in psychological and perceptual variables rather than in demographic characteristics (Table 9 and Table 10). In particular, the chi-square results revealed significant differences in psychological variables, with clear variation across clusters in food neophobia and especially strong differences in concern for food safety (Table 9). No significant differences were found between clusters in terms of gender, place of residence, field of study, or economic status, which is also reflected in the very low values of Cramér’s V (<0.06) (Table 9). This indicates that sociodemographic factors did not play a significant role in differentiating the identified segments.

The results of the analysis of variance (Table 10) revealed very large and statistically significant differences between clusters across all three latent dimensions identified in the EFA. These differences were associated with large effect sizes (η^2^ ranging from 0.756 to 0.793), confirming that the key criterion for segmentation was the perception and acceptance of specific types of products rather than classical demographic variables.

### 4.2. Consumer Segmentation Based on EFA Factors Related to Product Attribute Preferences for Insect-Based Foods

Consumer segmentation was conducted based on the results of the exploratory factor analysis (EFA), which identified three latent dimensions describing the perception of attributes of foods containing edible insects: (1) sensory–economic, (2) practical–market, and (3) informational–health. Using the factor scores, a k-means cluster analysis was performed. Cluster sizes were well balanced: Cluster 1 included 267 respondents (28.19%), Cluster 2 included 365 (38.55%), and Cluster 3 included 315 (33.26%). The high reproducibility of the solution was confirmed in subsampling analyses: the Adjusted Rand Index (ARI) across 100 random 80% subsamples averaged 0.98 (SD = 0.01; 2.5–97.5% quantiles: 0.95–1.00), indicating high stability of the cluster assignment. For k = 3, the within-cluster sum of squares (WSS = 609.8) relative to the total scatter (TSS = 2841.0) yielded a proportion of variance explained of PVE = 0.79, demonstrating that the model accounted for a substantial share of variability in the data. Additional validity indices supported the chosen solution (Silhouette = 0.46, Calinski–Harabasz = 1726.9, Davies–Bouldin = 0.87). Taken together, these results indicate that the three-cluster solution was both statistically robust and interpretable in terms of consumer profiles.

#### 4.2.1. Cluster 1—Quality-Oriented Pragmatists

Respondents in this cluster obtained positive scores on the sensory–economic dimension, values close to zero on the practical–market dimension, and negative scores on the informational–health dimension (Table 11). Psychologically, they were characterized by a moderate level of food neophobia (NF = 2.27), a moderate level of concern for the environment, and elevated concern for food safety (Table 12). The gender distribution was balanced (Table 13). Respondents lived both in rural areas (34.70%) and in large cities with more than 150,000 inhabitants (29.34%). The majority reported a moderate economic status (54.57%). In terms of academic profile, 49.21% were students of social and humanities sciences (Table 13).

#### 4.2.2. Cluster 2—Skeptical and Disengaged

Respondents in this segment obtained the lowest scores across all factor dimensions (Table 11). Psychologically, this group exhibited the highest level of food neophobia (NF = 2.58) and the lowest levels of concern for both food safety and the environment, across the clusters (Table 12). Women accounted for 58.80% of this cluster (Table 13). The largest proportion of respondents came from rural areas (44.57%). A majority reported a moderate economic status (51.69%). With regard to field of study, 44.57% were students of engineering and technical sciences (Table 13).

#### 4.2.3. Cluster 3—Conscious Enthusiasts

Respondents in this cluster achieved the highest scores across all factor dimensions (Table 11). This was the group most open to the attributes of insect-based foods, whether sensory, practical, or informational–health. They were characterized by the lowest level of food neophobia (NF = 1.98), the highest concern for food safety (CFS = 3.82), and strong pro-environmental attitudes (Table 12). Women constituted 68.04% of this cluster (Table 13). The place of residence was evenly divided between rural areas (33.06%) and large cities (33.88%). Most respondents declared a moderate economic status (52.62%). In terms of academic profile, 41.05% were students of social and humanities sciences and 41.05% of engineering and technical sciences (Table 13).

#### 4.2.4. Factors Differentiating Attribute-Based Clusters

Chi-square tests and ANOVA confirmed that the most pronounced differences between clusters were found in psychological and perceptual variables, as well as in selected demographic characteristics (Table 14 and Table 15). The chi-square results indicated clear differences in food neophobia, with particularly strong effects observed for concern for food safety and concern for the environment, as reflected in high values of Cramér’s V (>0.43) (Table 14). Among demographic variables, significant differences were noted only for gender (*p* < 0.001, V = 0.134) and place of residence (*p* = 0.009, V = 0.076), although the strength of these associations was small. Field of study and economic status did not significantly differentiate the clusters (Table 14). This suggests that psychological and perceptual factors were far more important determinants of segmentation than classical sociodemographic variables.

The results of the analysis of variance (Table 15) confirmed very strong and statistically significant differences between clusters across all three latent dimensions identified in the EFA. The high η^2^ values (0.783–0.790) indicate a large effect size and confirm that the key criterion for segmentation was the perception and acceptance of specific product attributes.

## 5. Discussion

The growing societal interest in sustainable consumption, particularly in the context of the climate crisis and the rising global population, prompted the authors to conduct a study aimed at identifying homogeneous clusters of young consumers in Poland with regard to edible insect consumption. The acceptance of edible insects as a regular part of the human diet aligns with the principles of sustainable food consumption. Insects have attracted the attention of researchers not only due to their potential to contribute to global food security, as they are a rich source of high-quality protein, but also because their production generates a low carbon footprint, requires less water and feed, and is more environmentally efficient compared to conventional livestock farming [41,42].

Despite ongoing marketing efforts emphasizing the health benefits of insects and the environmental advantages of insect farming, these campaigns have not succeeded in changing European public attitudes from negative to positive toward entomophagy. Food neophobia remains high, particularly among Western European populations [11,30]. Similarly, Polish citizens continue to show low willingness to incorporate insects into their diets [43]. Key barriers to the acceptance of edible insects among Polish consumers include food neophobia [13,44], feelings of disgust, lack of experience, and limited knowledge on how to prepare insect-based foods in everyday meals [44]. On the other hand, it was found that among both younger and older consumers in Poland, the overall acceptance of soups containing edible insects was primarily influenced by taste and texture [45]. According to the authors, the observed trend of increasing acceptance in both age groups suggests that edible insects may eventually become an accepted element of a sustainable diet within Polish society. The literature highlights that young consumers from Generation Z, such as university students, represent a demographic group with significant potential to transform consumption patterns and adopt innovative solutions [27]. Research has shown that Polish students’ decisions to consume insect-based foods are influenced by three main and independent categories of factors, health and environmental awareness, organoleptic qualities, and dietary habits [13]. It has also been confirmed that the greater the students’ awareness of health and environmental issues, the more likely they are to consider trying foods containing edible insects [12,35]. Furthermore, previous studies have demonstrated that a positive attitude toward edible insects, as well as an intention to purchase insect-based products, is strongly correlated with declared willingness to consume them [12]. International research points to a range of additional moderating variables that influence consumers’ attitudes toward insects. Beyond health awareness, environmental concern, and food neophobia, factors such as gender, familiarity with entomophagy, ethical beliefs, the visual absence of insects in food products, and prior consumption experience have all been shown to affect the acceptance of edible insects and insect-based foods among young consumers, including students [30,46].

Our study confirmed that Polish university students from Generation Z place strong emphasis on information provided by food producers concerning the health and nutritional value of edible insect-based foods. In addition, the analysis identified two segmentation solutions: a four-cluster model based on product categories and a three-cluster model derived from product attributes. In the four-cluster solution, the segments were labeled insect product enthusiasts, pragmatic consumers of processed products, cautious explorers, and skeptical and disengaged. In the three-cluster solution, the segments were identified as conscious enthusiasts, quality-oriented pragmatists, and skeptical and disengaged. Across both approaches, the clusters differed significantly in terms of attitudes toward insect-based foods, food neophobia, and perceptions of sustainability. However, no statistically significant differences were observed with respect to socio-demographic characteristics such as gender, place of residence, field of study, or economic status. Notably, food neophobia emerged as a strong differentiator between clusters (*p* < 0.001), confirming its central role as a psychological barrier to the acceptance of insect-based foods. The segmentation into distinct consumer profiles aligns with previous findings in the literature. Our results support earlier studies. For example, Kamenidou et al. [47] segmented Generation Z consumers into four homogeneous groups: “Future potential insect consumers” (29.1%), “Rejecters” (26.7%), “Disgusted, prefer to starve” (22.2%), and “Inconsistent” (22.0%). Similarly, Brunner and Nuttavuthisit [48], in their study on Swiss consumers, found that only around 9% could be classified as early adopters of insect-based foods, while the majority held skeptical or rejecting attitudes. Puteri et al. [49] also described comparable consumer segments in Germany, among which only one clearly rejected insect-based products. Importantly, they found that most consumers prioritized product naturalness over environmental or nutritional benefits. Furthermore, trust in institutions and food safety emerged as crucial factors for skeptical consumers, an observation consistent with our own findings in the cautious explorers cluster.

One of the factors that strongly and significantly differentiated the clusters of young Generation Z consumers in our study was food neophobia (*p* < 0.001). The role of food neophobia in limiting the acceptance of insect-based products has been well documented in previous research [50,51], which found a significant negative impact of food neophobia on attitudes toward eating insects. Consistent with these findings, our results show that respondents with lower levels of food neophobia, specifically those classified within the pragmatic consumers of processed products and cautious explorers clusters, were more likely to accept products in which the presence of insects was not visually apparent.

Sensory attributes such as taste, smell, and the attractive appearance of products containing edible insects proved important in segmenting young Generation Z consumers. These results are consistent with previous research highlighting the role of sensory appeal in the acceptance of novel foods. Notably, insect-based products in which insects are not visually identifiable tend to generate greater consumer interest [33,50].

An interesting observation emerging from our study was the moderate influence of environmental motivations on young consumers’ willingness to try insect-based foods. Only respondents in cluster 3 (conscious enthusiasts), the group most open to insect-based foods, demonstrated strong pro-environmental attitudes while also expressing notable concerns about food safety. Although the Quality-Oriented Pragmatists cluster showed a high level of environmental concern, “trendiness” or social fashion was rated as a relatively unimportant factor in their consumption choices. This aligns with the findings of Puteri et al. [49], who noted that consumers tend to prioritize naturalness and food safety over environmental benefits.

Finally, our results highlight the importance of adapting communication and marketing strategies to the specific characteristics of each identified consumer segment. The conscious enthusiasts cluster includes consumers already well-informed about sustainable consumption; this group may be effectively reached through direct and transparent product communication. In contrast, the quality-oriented pragmatists respond best to messaging that emphasizes taste and functional benefits of insect-based foods. The cautious explorers cluster would benefit most from public sector involvement in educational and informational campaigns aimed at building trust in the safety of novel foods. Meanwhile, the skeptical and disengaged cluster appears to hold the least potential in advancing the idea of sustainable consumption, reinforcing earlier findings that certain consumer groups remain largely resistant to conventional entomophagy promotion strategies [50].

Our findings confirm the central role of psychological factors, particularly food neophobia, concern for food safety, and environmental sustainability, in shaping consumer acceptance of insect-based foods, in line with the broader literature. For example, Verbeke [27], Schlup and Brunner [52], Palmieri et al. [53], and Lammers [54] showed that higher food neophobia is strongly associated with lower willingness to consume insect-based products, reinforcing its interpretation as a major barrier. Similarly, Abbasi et al. [55] identified cultural norms, feelings of disgust, and perceived health risks as critical obstacles to entomophagy in European contexts.

With regard to segmentation based on product attributes, Puteri et al. [49] also identified distinct consumer profiles, showing that preferences vary considerably depending on sensory and informational features. This supports our finding that perceptual dimensions are more influential than sociodemographic characteristics in defining consumer clusters.

A wide range of statistical analyses confirmed that, when segmenting young consumers, specifically Generation Z students in Poland, the key criteria were perceptions of product attributes (e.g., sensory, health-related, informational, economic, and market-practical characteristics) and the acceptance of specific product types, rather than demographic characteristics. Classic demographic variables such as gender, place of residence, field of study, and economic status did not significantly differentiate the identified segments. This applied to both segmentation approaches: clustering based on the propensity to consume products from 11 categories and clustering based on the perception of product characteristics. We confirmed that a strong and statistically significant criterion for segmentation was the perception of, and attitudes toward, specific insect products or products containing hidden insect ingredients. Among the three consumer profiles identified, conscious enthusiasts (cluster 3) are likely to play the most important role in supporting the acceptance of entomophagy in Poland. By contrast, quality-oriented pragmatists (cluster 1) are expected to play a smaller role, while skeptical and disengaged (cluster 2) may not contribute to this process at all.

Systematic reviews provide further evidence. Alhujaili et al. [33] and Mina et al. [17] emphasized a broad set of factors influencing acceptance of insect-based foods, ranging from individual characteristics (e.g., neophobia, familiarity) to marketing mix elements (product, price, promotion, and place). These frameworks resonate with our integrated segmentation approach and highlight the importance of combining psychological, perceptual, and sociodemographic dimensions in consumer profiling.

Finally, studies conducted in different cultural contexts, such as exploratory research in Italy [56] and cross-country comparisons between Switzerland and Thailand [48], point to similar constructs, neophobia, sustainability, and product perception, in shaping consumer attitudes toward edible insects. Complementing these findings, evidence from a representative study in Poland shows that while insects may be accepted as a general meat alternative, this does not necessarily translate into willingness to purchase and consume them [57]. Taken together, this body of evidence indicates that our results are consistent with prior research while also providing new insights that advance current understanding in the field.

## 6. Conclusions

This study demonstrated that young consumers can be meaningfully segmented based on their perceptions of insect-based foods. Two complementary clustering approaches were applied: one based on willingness to consume products across 11 categories, which yielded a four-cluster solution, and another based on perceived importance of product attributes, which resulted in three clusters. Despite these differences in methodological scope, both approaches pointed to the same conclusion: psychological factors (food neophobia, concern for food safety, and concern for environmental sustainability) and perceptual evaluations of product attributes (e.g., taste, aroma, health and nutrition claims) were the strongest determinants of consumer acceptance, whereas sociodemographic variables played only a marginal role. Importantly, using factor analysis prior to clustering allowed us to capture latent dimensions of perception, providing a more stable and interpretable basis for identifying consumer profiles than raw mean ratings.

From a theoretical perspective, the findings extend the literature on sustainable food consumption and entomophagy by showing that robust latent dimensions can reveal meaningful consumer heterogeneity. Methodologically, the integration of factor analysis with cluster analysis offers a rigorous framework for studying consumer segmentation in emerging food markets.

From a practical standpoint, the identified clusters provide valuable guidance for developing targeted communication and marketing strategies. Segments characterized by curiosity but lingering uncertainty, such as cautious explorers, could be addressed through educational initiatives and transparent information, while more open groups, such as conscious enthusiasts, may be engaged through messages emphasizing sustainability and food safety. Such tailored strategies could help accelerate the normalization of insect-based products among younger generations.

Overall, the results highlight the need for consumer education and marketing efforts that are adapted to distinct psychological and perceptual profiles, rather than relying on demographic categories. This has important implications not only for the food industry but also for policymakers and educators seeking to promote sustainable dietary shifts in Generation Z populations.

### Limitations

Despite the comprehensive analytical scope, this study is subject to several limitations. First, the sample was limited to young adults, primarily university students, which may constrain the generalizability of the findings to the broader population. Moreover, as participation was voluntary and the total number of students invited as well as the response rate were not recorded, the possibility of self-selection bias cannot be excluded. It is plausible that individuals with greater interest in food innovation and entomophagy were more willing to participate in the survey, while those with more skeptical attitudes chose not to respond. This should be taken into account when interpreting the results. Second, the data relied on self-reported declarations rather than observed purchasing or consumption behavior, which may introduce social desirability bias or a gap between intentions and actual actions. Additionally, the psychometric instruments used to assess perceptions and attitudes (e.g., food neophobia scale) were based on self-assessment, making them susceptible to situational influences or socially desirable responding. Finally, although the analysis included a wide range of product-related attributes, other potentially influential factors, such as cultural norms, religious beliefs, or prior experiences with alternative foods, may also shape consumer acceptance of edible insects and should be explored in future research. Future studies should also validate these findings on more representative samples of the general population, and comparative cross-cultural research could provide additional insights into differences and similarities across consumer groups.

## Figures and Tables

**Table 1:** Characteristics of the respondent group.

Variable	N	%
Gender		
Female	568	59.98
Male	379	40.02
Field of study		
Engineering and technical sciences	389	41.08
Social sciences	382	40.34
Medical and health sciences	74	7.81
Natural and physical sciences	48	5.07
Humanities	30	3.17
Agricultural sciences	24	2.53
Place of residence		
Rural area	349	36.85
Town (up to 50,000 inhabitants)	167	17.63
Town (50,000–150,000 inhabitants)	157	16.58
City (150,000–500,000 inhabitants)	208	21.96
City (>500,000 inhabitants)	66	6.97
Self-declared economic status		
Insufficient	27	2.85
Sufficient	215	22.7
Good	502	53.01
Very good	198	20.91
Exceptionally high economic status	5	0.53

**Table 2:** Variance explained by the three-factor solution of food product categories.

Factors	SS Loadings	% of Variance	Cumulative %
F1 *	4.63	42.1	42.1
F2	2.87	26.1	68.2
F3	1.64	14.9	83.1

* F1—Conventional food products; F2—Insect-based products; F3—Dairy and culinary add-ons.

**Table 3:** Factor loadings of food products containing edible insects.

Product	Fa1 *	F2	F3
Burgers and processed meat products	0.844		
Ready-to-eat meals (soups, pasta, pancakes, sauces)	0.931		
Bakery products (bread, rolls, pizza, etc.)	0.917		
Confectionery (cakes, cookies, chocolate-coated insects, desserts)	0.809		
Snacks (bars, crisps)	0.890		
Post-workout beverages and high-protein supplements	0.497		
Dairy-based products (e.g., cottage cheese, yogurts)			0.982
Sauces and mayonnaise			0.460
Cricket (frozen, dried, or powdered form)		0.897	
Mealworm larvae (frozen, dried, or powdered form)		0.950	
Migratory locust (frozen, dried, or powdered form)		0.981	

* F1—Conventional food products; F2—Insect-based products; F3—Dairy and culinary add-ons.

**Table 4:** Variance explained by the three-factor solution of edible insect food attributes.

Factors	SS Loadings	% of Variance	Cumulative %
F1 *	3.96	30.4	30.4
F2	3.55	27.3	57.7
F3	2.85	21.9	79.6

* F1—Sensory-economic, F2—Practical-market, F3—Informational-health.

**Table 5:** Factor loadings for attributes of foods containing edible insects.

Product Attribute	F1 *	F2	F3
Appealing taste	0.922		
Pleasant aroma	0.933		
Attractive appearance	0.842		
High nutritional value			0.400
Product variety and market availability		0.535	
Popularity (trendiness)		0.716	
Affordable price	0.478		
Package size and attractiveness		0.669	
Ease of use		0.523	
Health claims			0.932
Nutrition claims			0.933
Reduction in CO_2_ emissions		0.561	
Recipe availability on blogs and websites		0.754	

* F1—Sensory-economic, F2—Practical-market, F3—Informational-health.

**Table 6:** Mean factor scores from EFA for product categories across consumer clusters.

Factor	C1 **	C2	C3	C4
	Mean ± SE			
F1 *	0.86 ± 0.04	−0.96 ± 0.02	1.15 ± 0.04	0.09 ± 0.02
F2	−0.36 ± 0.04	−0.85 ± 0.01	1.60 ± 0.05	0.24 ± 0.04
F3	1.02 ± 0.05	−0.92 ± 0.01	1.33 ± 0.05	−0.09 ± 0.02

* F1—Conventional food products; F2—Insect-based products; F3—Dairy and culinary add-ons; ** C1—Pragmatic consumers of processed products, C2—Skeptical and disengaged, C3—Insect product enthusiasts, C4—Cautious explorers.

**Table 7:** Mean values of psychological variables across product-based consumer clusters.

Cluster	NF **	CFS	CES
	Mean ± SE		
C1 *	1.89 ± 0.007	3.73 ± 0. 08	3.56 ± 0.08
C2	2.56 ± 0.05	2.42 ± 0.07	2.31± 0.06
C3	1.99 ± 0.06	4.09 ± 0.07	4.03 ± 0.06
C4	2.19 ± 0.04	3.33 ± 0.05	3.15 ± 0.05

* C1—Pragmatic consumers of processed products, C2—Skeptical and disengaged, C3—Insect product enthusiasts, C4—Cautious explorers; ** NF—Food neophobia, CFS—Concern for food safety, CES—Concern for environmental sustainability.

**Table 8:** Sociodemographic characteristics of respondents across product-based clusters.

Cluster	C1 *	C2	C3	C4	Global χ^2^ *p*
	%				
Men	33.86	37.05	43.87	43.54	0.321
Women	66.14	62.95	56.13	56.46	
Rural area	33.86	38.86	33.55	37.54	0.818
Town (up to 50,000 inhabitants)	19.69	17.17	18.06	17.12	
Town (50,000–150,000 inhabitants)	14.17	18.37	17.42	15.32	
City (>150,000 inhabitants)	32.28	25.6	30.97	30.03	
Lower economic status	18.9	25.6	29.68	26.13	0.308
Moderate economic status	59.84	50.3	50.32	54.35	
Higher economic status	21.26	24.1	20	19.52	
Engineering and technical sciences	38.58	40.96	41.94	41.74	0.116
Natural sciences	15.75	15.06	21.29	12.91	
Social and humanities sciences	45.67	43.98	36.77	45.35	

* C1—Pragmatic consumers of processed products, C2—Skeptical and disengaged, C3—Insect product enthusiasts, C4—Cautious explorers.

**Table 9:** Results of chi-square tests for demographic and psychological variables across product-based clusters.

Variable	χ^2^	df	*p*-Value	Cramér’s V
Gender	5.910	3	0.116	0.055
Field of study	7.143	6	0.308	0.025
Place of residence	5.183	9	0.818	0.000
Self-declared economic status	6.997	6	0.321	0.023
NF *	87.500	12	<0.001	0.163
CFS	365.889	12	<0.001	0.353
CES	5.910	3	0.116	0.055

* NF—Food neophobia, CFS—Concern for food safety, CES—Concern for environmental sustainability.

**Table 10:** Results of ANOVA for EFA factor scores across product-based clusters.

Factor	SS (Between)	df (Between)	SS (Within)	df (Within)	F	η^2^	*p*-Value
F1 *	610.228	3	159.685	943	1201.207	0.793	<0.001
F2	668.645	3	216.387	943	971.304	0.756	<0.001
F3	688.389	3	194.676	943	1111.508	0.780	<0.001

* F1—Conventional food products; F2—Insect-based products; F3—Dairy and culinary add-ons.

**Table 11:** Mean factor scores from EFA for product attributes across consumer clusters.

Factor	C1 **	C2	C3
	Mean ± SE		
F1 *	0.20 ± 0.03	−1.29 ± 0.03	0.77 ± 0.02
F2	−0.02 ± 0.01	−1.07 ± 0.02	0.80 ± 0.02
F3	−0.11 ± 0.02	−1.19 ± 0.02	0.97 ± 0.02

* F1—Sensory-economic, F2—Practical-market, F3—Informational-health; ** C1—Quality-oriented pragmatists, C2—Skeptical and disengaged, C3—Conscious enthusiasts.

**Table 12:** Mean values of psychological variables across attribute-based consumer clusters.

Cluster	NF **	CFS	CES
	Mean ± SE		
C1 *	2.27 ± 0.04	3.40 ± 0.05	3.13 ± 0.05
C2	2.58 ± 0.05	2.08 ± 0.05	2.05 ± 0.05
C3	1.98 ± 0.04	3.82 ± 0.05	3.72 ± 0.04

* C1—Quality-oriented pragmatists, C2—Skeptical and disengaged, C3—Conscious enthusiasts; ** NF—Food neophobia, CFS—Concern for food safety, CES—Concern for environmental sustainability.

**Table 13:** Sociodemographic characteristics of respondents across attribute-based clusters.

Cluster	C1 *	C2	C3	Global χ^2^ *p*
	%			
Men	48.26	41.2	31.96	0.134
Women	51.74	58.8	68.04	
Rural area	34.7	44.57	33.06	0.076
Town (up to 50,000 inhabitants)	19.24	14.98	18.18	
Town (50,000–150,000 inhabitants)	16.72	18.73	14.88	
City (>150,000 inhabitants)	29.34	21.72	33.88	
Lower economic status	26.18	23.22	26.72	<0.001
Moderate economic status	54.57	51.69	52.62	
Higher economic status	19.24	25.09 **	20.66	
Engineering and technical sciences	38.17	**44.57**	41.05	0.047
Natural sciences	12.62	15.36	17.91	
Social and humanities sciences	49.21	40.07	41.05	

* C1—Quality-oriented pragmatists, C2—Skeptical and disengaged, C3—Conscious enthusiasts; ** Bold values indicate proportions significantly different from the rest of the sample (*p* < 0.05, chi-square tests with Benjamini–Hochberg correction).

**Table 14:** Results of chi-square tests for demographic and psychological variables across attribute-based clusters.

Variable	χ^2^	df	*p*-Value	Cramér’s V
Gender	18.966	2	<0.001	0.134
Field of study	8.114	4	0.087	0.047
Place of residence	16.996	6	0.009	0.076
Self-declared economic status	3.536	4	0.472	0.000
NF *	81.193	8	<0.001	0.197
CFS	384.23	8	<0.001	0.446
CES	370.521	8	<0.001	0.438

* NF—Food neophobia, CFS—Concern for food safety, CES—Concern for environmental sustainability.

**Table 15:** Results of ANOVA for EFA factor scores across attribute-based clusters.

Factor	SS (Between)	df (Between)	SS (Within)	df (Within)	F	η^2^	*p*-Value
F1 *	673.502	2	179.487	944	1771.118	0.790	<0.001
F2	536.722	2	147.992	944	1711.796	0.784	<0.001
F3	720.277	2	200.116	944	1698.867	0.783	<0.001

* F1—Sensory-economic, F2—Practical-market, F3—Informational-health.

## Data Availability

All relevant data for this study are publicly available from the Github repository (https://github.com/ResearchData-Articles/Segmentation-of-Young-Polish-Consumers). uploaded on 10 September 2025.

## Supplementary Materials

The following supporting information can be downloaded at https://www.mdpi.com/article/10.3390/insects16090980/s1. S1 Survey Questions
