# Supplementary material for: Segmentation of Young Polish Consumers in Relation to Product Attributes and Willingness to Consume Food Containing Edible Insects
Source: Insects. 2025 Sep 19;16(9):980. doi: 10.3390/insects16090980 (PMC12470507; doi:10.3390/insects16090980)
Supplement: Supplementary file 1 [file insects-16-00980-s001.zip › insects-3752336-supplementary.pdf]

Survey Questions

Part 1. Metrics

Gender:
 female
 male
 non-binary or gender diverse
 I prefer not to disclose

Age (in completed years): ……………..

Study profile
☐ Engineering and technical sciences
☐ Social science
☐ Medical and health science
☐ Natural and physical sciences
☐ Humanities
☐ Agricultural science

Place of residence
 Rural area
 Town (up to 50,000 inhabitants)
 Town (50,000–150,000 inhabitants)
 City (150,000–500,000 inhabitants)
 City (>500,000 inhabitants)

How do you assess your financial situation?
 insufficient
 sufficient
 good
 very good
 exceptionally high economic status

Survey part 2

1 To what extent do you agree with the following statements
(Statements on attitudes toward "new foods" - Food Neophobia Scale)
Statements:
1.1. I constantly sampling new and differentiated foods.
1.2. I do not trust new foods.
1.3. If I don't know what is in a food, I won't try it.
1.4. I like foods from different cultures.
1.5. Ethnic food looks too weird to eat.
1.6. At dinner parties, I will try new foods.
1.7. I am afraid to eat things I have never had before.
1.8. I am very particular about the foods I eat..
1.9. I will eat almost everything.
1.10. I like to try new ethnic restaurants.

Answers:
definitely not
rather not
don't know, don't have an opinion/ neither yes nor no
rather yes
definitely yes

2 In your opinion, can the practice of consuming food containing edible insects (fresh, frozen, dried, powdered e.g. meal) solve problems such as:
(Concern for food safety: 2.1-2.4 items, and Concern for environmental sustainability: 2.5-2.6 items)
Statements:
2.1 Hunger and malnutrition
2.2. Ensuring access to food with high nutritional value (food security)
2.3. High demand for protein sources
2.4. Ensure access to foods with high concentrations of n-3 fatty acids
2.5 Sustainability
2.6. Reducing greenhouse gas emissions

Answers:
definitely not
rather not
don't know/don't have an opinion
rather yes
definitely yes

3. If a "new innovative food" containing edible insects (fresh, frozen, dried, powdered e.g. meal) appears on the market, what factors could influence you to try it:
Statements:
3.1. Attractive taste
3.2. attractive smell
3.3. Attractive appearance
3.4. High nutritional value
3.5. Varied assortment and availability for sale
3.6. Popularity (fashion)
3.7. Affordable price
3.8 Size and attractiveness of packaging
3.9 Convenience of use
3.10. Health claims
3.11. Nutritional claims
3.12. Reduction of CO_2 emissions
3.13. Availability of recipes on blogs and websites

Answers:
definitely not
rather not
don't know, don't have an opinion
rather yes
definitely yes

4 Do you express a willingness to consume "new foods" containing edible insects (fresh, frozen, dried, powdered e.g. meal) if they were available in the form of the following products?

Products:

4.1 Hamburgers, meat products and preparations
4.2 Prepared meals: soups, pasta, pancakes, sauces, etc.
4.3 Bread, rolls, pizza, and other bakery products
4.4 Cakes, cookies, chocolate-covered insects, other pastry and confectionery products
4.5 Bars, Chips, and other snacks
4.6 Post-workout drinks, nutritional supplements for people with high protein requirements
4.7 Cottage cheese, yoghurt and other fermented dairy products
4.8 Sauces, mayonnaise
4.9. Crickets (in frozen, dried or powdered form)
4.10. Mealworm larvae (in frozen, dried or powdered form)
4.11. Migratory locusts (in frozen, dried or powdered form)
